# Stress echocardiography in valvular heart disease

**DOI:** 10.3389/fcvm.2023.1233924

**Published:** 2023-12-14

**Authors:** Kensuke Hirasawa, Masaki Izumo, Yoshihiro J. Akashi

**Affiliations:** ^1^ Department of Cardiovascular Medicine, Tokyo Medical and Dental University, Tokyo, Japan; ^2^ Division of Cardiology, Department of Internal Medicine, St. Marianna University School of Medicine, Kawasaki, Japan

**Keywords:** valvular heart disease, stress echocardiography, exercise, aortic stenosis, mitral regurgitation

## Abstract

Valvular heart disease (VHD) has been a significant health problem, particularly in developed countries, in relation to the aging population. Recent developments in the management of VHD require a more accurate assessment of disease severity to determine the need for transcatheter interventions or open heart surgery. Stress echocardiography is a crucial imaging modality for identifying the underlying pathology of VHD. Optimal administration of exercise or intravenous drugs may reveal hemodynamic abnormalities under stress without posing an invasive risk. Therefore, the implementation of stress echocardiography is recommended for determining interventional indications and risk stratification in mitral regurgitation and aortic stenosis. In addition, recent evidence has accumulated regarding the usefulness of stress echocardiography in various conditions including mitral stenosis, aortic regurgitation, and post-interventional VHD. Here, we summarize the current evidence and future perspectives on stress echocardiography in VHD.

## 1. Introduction

Valvular heart disease (VHD) is regarded as a major health problem worldwide. Since the epidemiology changes from rheumatic to degenerative, the prevalence of VHD increases as the population ages especially in developed countries (1, 2). In recent decades, treatment options for VHD are getting varied with the development of surgical and transcatheter techniques. Getting higher the ages of patients who are indicated for VHD intervention, a more accurate assessment of disease severity and risk stratification is warranted. Imaging modalities including three-dimensional echocardiography, computed tomography, and magnetic resonance imaging have been highly revolutionized with the improvement of the scanners and quantification software. Although those techniques provide accurate functional and anatomical data derived by three-dimensional reconstruction with high spatial resolution, only limited information can be obtained on hemodynamic changes in stress conditions.

Stress echocardiography (SE) had first established for diagnosing and stratifying the risk of ischemic heart disease by assessing wall motion abnormality (3). SE has also recently been used for hemodynamic assessment during the stress of various heart diseases. In particular, the evidence in VHD has rapidly accumulated and the latest guidelines recommend implementation of SE in the decision-making for the treatment of aortic stenosis (AS) and mitral regurgitation (MR) (4, 5). Moreover, several novel insights on the other VHD have been continuously investigated.

To utilize SE in clinical practice, it is essential to know the optimal indication, the utility, and the limitation. The stress protocol, including exercise and drug infusion, varies and should be selected properly for each pathology. In addition, data acquisition during stress is highly technical in some case. Therefore, both physicians and sonographers are required to be well experienced with SE.

In this review, we summarize current knowledge on SE for VHD and discuss the future perspectives.

## 2. Stress protocols and contraindications of stress echocardiography

SE for VHD is performed with several stress modalities such as exercise (treadmill or supine cycle ergometer) or pharmacological stress represented by dobutamine infusion. The indications for SE and the parameters to be measured are summarised in Table 1.

**Table 1:** Indications, stress protocols and parameters recommended for measurement of SE according to etiologies of VHD.

	Indication	Stress protocol	Parameters
AS	Classical LFLG severe AS	Dobutamine	Mean PG, FR, SV, and LV systolic reserve
			AVA_proj_ should be calculated
	Asymptomatic severe AS	Exercise	Symptoms during exercise, mean PG, AVA, and presence of Ex-PH
	Equivocal symptomatic moderate AS		
			(Zva, GLS, and FR during exercise may be useful)
MR	Primary MR	Exercise	Symptoms during exercise, increase of MR, and presence of Ex-PH
			(LV (LVEF and GLS) and RV (TAPSE) contractile reserve may be useful)
	Secondary MR	Exercise	Symptoms during exercise, increase of MR, and presence of Ex-PH
MS	Rheumatic MS	Exercise	Increase in mean transmitral PG and presence of Ex-PH
	Degenerative MS	Not established	Not established
Post-operative conditions	Suspected PPM	Exercise or dobutamine	Transprosthetic PG, EOA, DVI, and LV systolic reserve
	prosthesis degeneration		
	Mitral annuloplasty	Exercise or dobutamine	Transmitral PG and Ex-PH
Others	AR	Exercise	Not established
			(LV contractile reserve, GLS, ESV, and TAPSE, may be useful)
	TR	Not established	Not established

AR, aortic regurgitation; AS, aortic stenosis; AVA, aortic valve area; AVA_proj_, projected aortic valve area; DVI, Doppler velocity index; EOA, effective orifice area; ESV, end-systolic volume; Ex-PH, exercise induced pulmonary arterial hypertension; FR, flow rate; GLS, global longitudinal strain; LFLG, low-flow low-gradient; LV, left ventricle; MR, mitral regurgitation; MS, mitral stenosis; PG, pressure gradient; PPM, patient-prosthesis mismatch; TAPSE, tricuspid annular plane systolic excursion; TR, tricuspid regurgitation; Zva, valvulo-arterial impedance.

Close monitoring of ECG and blood pressure under the supervision of an experienced physician is recommended during exercise stress testing, especially in high-risk patients such as those with equivocal symptomatic AS. In the Bruce exercise protocol, which is the most frequently used protocol for both treadmill and cycle ergometer exercise testing, the patient begins with an initial workload of 25 W and the workload is increased by 25 W every 3 min in a stepwise manner. For patients with lower functional capacity and/or older patients, the ramp protocol can be applied, starting with a 10 W workload and gradually increasing 10 W every 3 min. Diagnostic endpoints of exercise stress testing are defined as follows; (i) symptoms, including severe chest pain, leg fatigue, dizziness, or other discomfort that limit continuous exercise, (ii) attainment of >85% of maximum predicted heart rate (MPHR), and (iii) attainment of a predefined specific workload.

As described in the following paragraph, pharmacological stress protocol using low-dose dobutamine infusion is applied in patients with low-flow low-gradient (LFLG) AS or post-operative valvular dysfunction. Usually the infusion is started with 5 µg/kg/min and increased by 2.5–5 µg/kg/min every 5 min up to 20 µg/kg/min for possible severe VHD and atropine infusion is not required for its safety. The diagnostic endpoints of dobutamine stress testing were (i) intolerable symptom development, (ii) attainment of >85% of MPHR, and (iii) attainment of maximal dose infusion, as well as exercise stress testing.

In both exercise and dobutamine stress testing, early termination of stress administration should be considered with the following conditions; abnormal response of vital signs, angina with significant ST changes on ECG, and ventricular/supraventricular arrhythmias.

Previous data from a large registry of SE reported that life-threatening events occurred in only 86 cases (0.1%) out of 85,997 examinations (6). While the safety of SE has been established, it is important to recognise several complications associated with SE and contraindications derived from stressors and/or disease characteristics.

The most common complications of exercise stress echocardiography (ESE) were arrhythmias (sustained/non-sustained ventricular tachycardia, supraventricular tachycardia, and atrioventricular block) and ischemic events (acute myocardial infarction and unstable angina) (6, 7). In addition, the potential risk of exercise-induced physical injury must be addressed to minimise the risk of mechanical complications (8). In addition to common contraindications to exercise testing such as unstable myocardial ischemia, uncontrolled arrhythmias, and severe symptomatic heart failure, it should be noted that symptomatic severe AS is a contraindication to ESE in the context of diagnosing VHD. Although dobutamine stress echocardiography (DSE), which is the most frequently performed pharmacological stress test for VHD, has similar contraindications to ESE, some additional contraindications need to be noted. Dobutamine infusion has been reported to be associated with arrhythmic complications and therefore be avoided in patients at high-risk of life-threatening arrhythmias. Furthermore, significant left ventricular obstruction, which is often comorbid in patients with AS, is also a contraindication to dobutamine.

## 3. Aortic stenosis

AS is characterized as a condition with narrowing of the aortic valve which induces restriction of forward output from left ventricle (LV). It is commonly known that the prognosis of patients with AS is still being poor as the disease severity advances. However, no medical treatment has yet shown promising data for preventing the disease progression (9). Therefore, current guidelines recommend surgical or transcatheter aortic valve replacement (AVR) in severe AS patients with symptoms and/or left ventricular ejection fraction less than 50% (4, 5). However, the clear diagnosis and management of AS with low LV output can be difficult since the true AS severity may be masked. Moreover, the optimal timing for invasive valvular intervention remains controversial in asymptomatic patients with AS.

### 3.1. Low-flow low-gradient (LF-LG) AS

**Figure 1:**
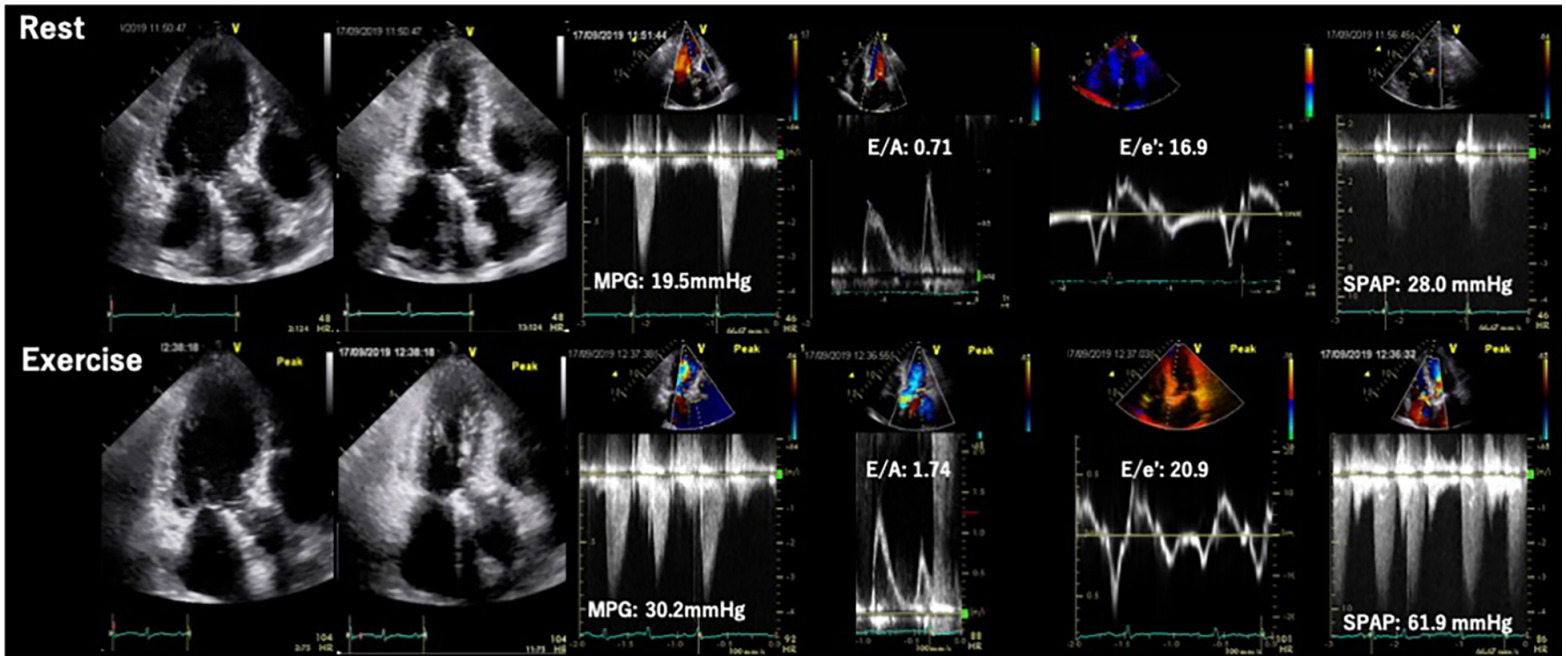
Classification of aortic stenosis according to stroke volume and mean transvalvular pressure gradient. AVA, aortic valve area; SVI, stroke volume index.

Determining the accurate disease severity of AS is sometimes challenging in the presence of low-flow state since the pressure gradient (PG) through the valve is directly related to the transvalvular flow. Therefore, the discrepancy between mean transvaluvular gradient and aortic valve area (AVA) may occur in patients with low LV output (LFLG severe AS) (10) (Figure 1). Patients with LV systolic dysfunction may have low mean transvaluvular gradient (<40 mmHg) despite having a calculated AVA of <1.0 cm^2^, a condition regarded as classical LF-LG severe AS (11, 12).

**Figure 2:**
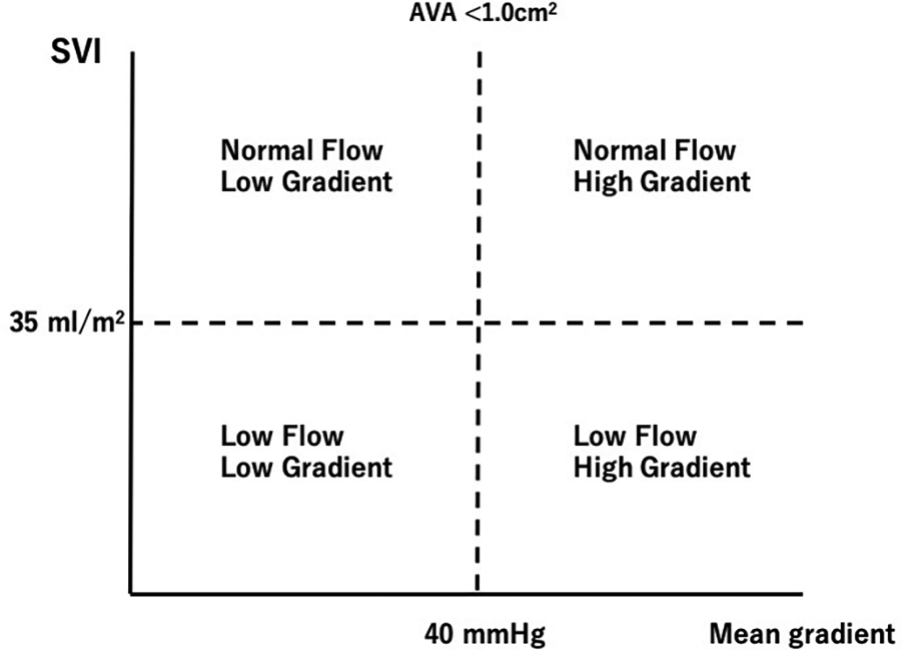
Evaluation of classical low-flow low-gradient aortic stenosis using dobutamine stress echocardiography. AS, aortic stenosis; AVA, aortic valve area; AVA_proj_, projected aortic valve area; EF, ejection fraction; FR, flow rate; PG, pressure gradient; SV, stroke volume.

**Figure 3:**
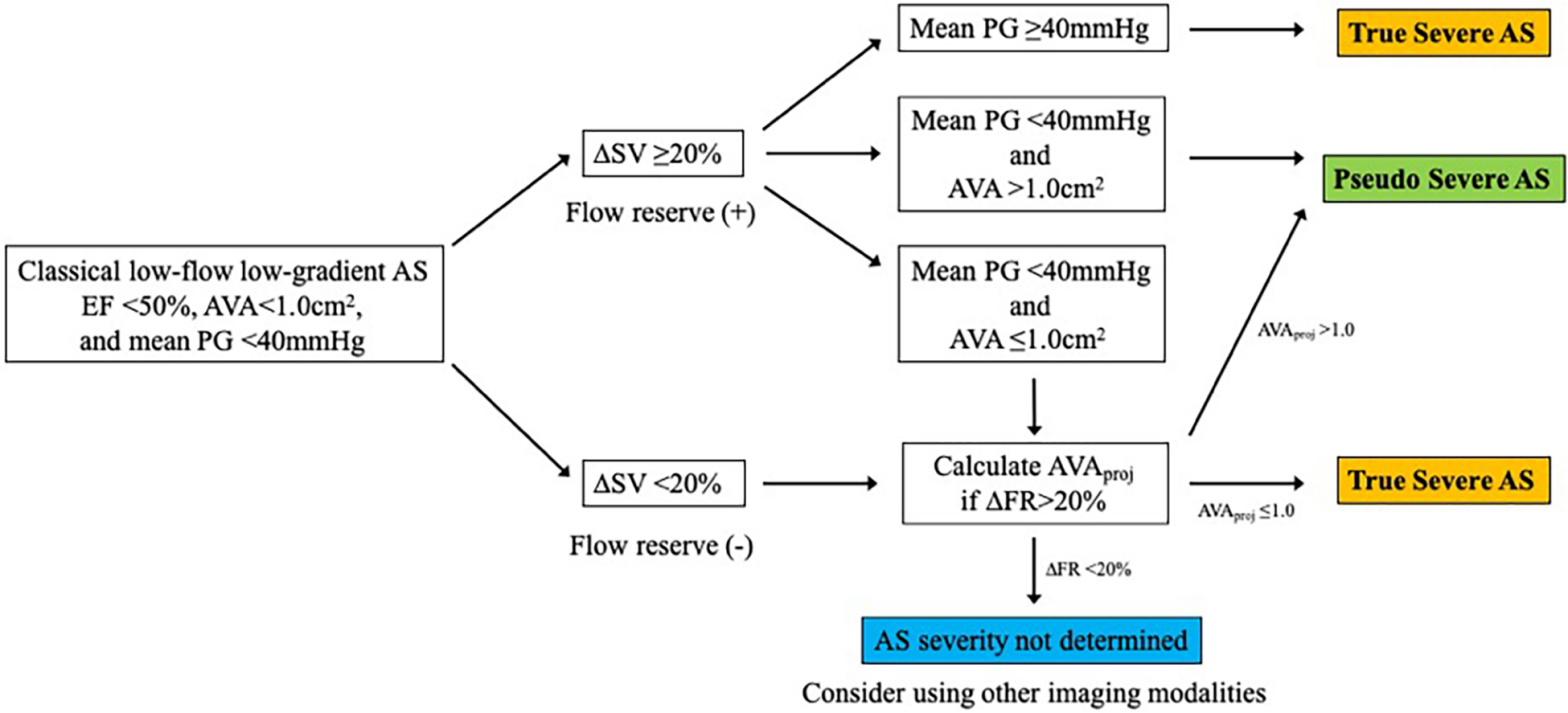
Pseudo severe AS case diagnosed by dobutamine stress echocardiography. A case of classical LFLG AS who underwent DSE. The SVI was significantly increased by low-dose dobutamine stress (20 γ) and the AVA increased by more than 1 cm^2^. The result confirmed pseudo severe AS in this case. AV, aortic valve; AVA, aortic valve area; DSE, dobutamine stress echocardiography; LFLG, low-flow low-gradient; LVOT, left ventricular outflow tract; MPG, mean pressure gradient; SVI, stroke volume index; VTI, time velocity integral.

SE is an essential imaging modality as it allows simultaneous assessment of the dynamic changes in flow condition and the disease severity. Low-dose DSE is currently recommended for symptomatic AS patients with transvalvular flow velocity of less than 4 m/s and LV ejection fraction (LVEF) less than 50% (i.e., classical LFLG severe AS, stage D2) (4, 5) (Figure 2). DSE is a powerful tool in identifying pseudo-severe AS caused by incomplete valve opening due to low flow condition. If the AVA increases by more than 1.0 cm^2^ in the presence of systolic flow reserve represented by a 20% increase in stroke volume (SV) during low-dose dobutamine administration, this may indicate that the disease severity was overestimated in assessment at rest (13) (Figure 3).

**Figure 4:**
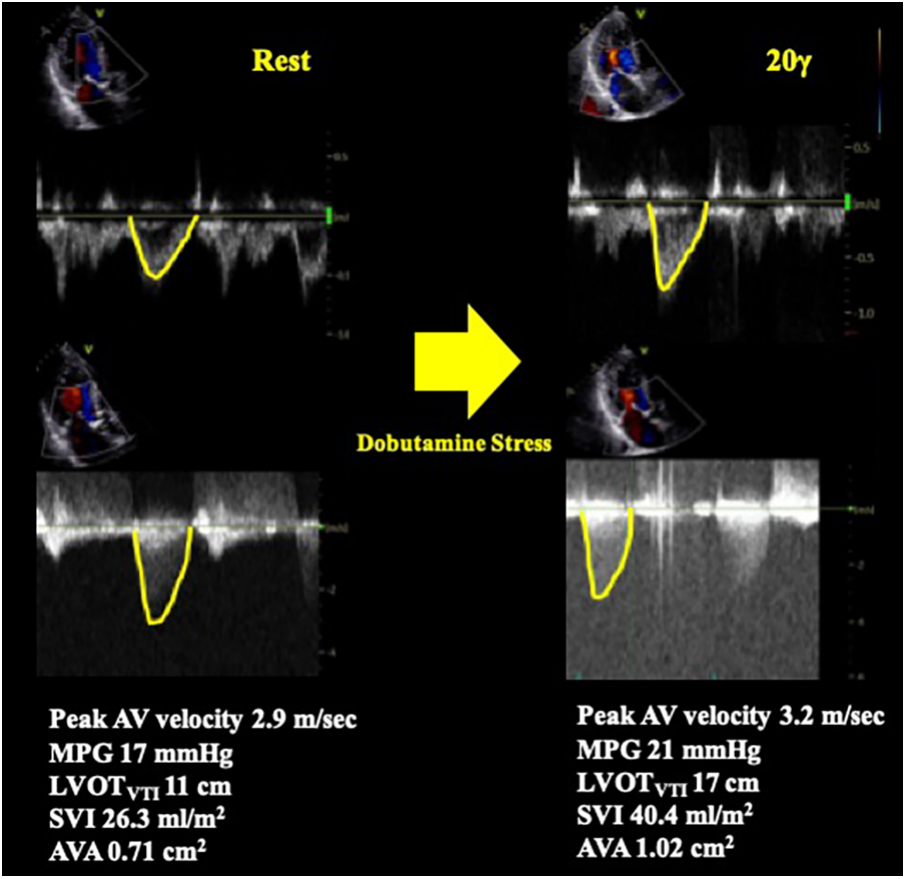
Calculation of projected aortic valve area with dobutamine stress echocardiography. AVA, aortic valve area; FR, flow rate; SV, stroke volume.

Pibarot P et al. proposed a quantitative method for evaluating AS severity using DSE (14). The projected AVA (AVA_proj_) represents the AVA calculated at a normal transvaluvlar flow rate (FR = 250 ml/sec) and is obtained from the relationship between FR and AVA with dobutamine loading using a linear regression equation. AVA_proj_ has high predictive values in discriminating true-severe AS from pseudo-severe AS (15) and provides an incremental prognostic value as an indicator for conservative medical treatment (16) (Figure 4).

Furthermore, DSE can also be applied to identify true-severe AS patients with moderate symptoms, as patients with AS commonly have multiple comorbidities such as lung disease or ischemic heart disease that may cause symptoms similar to AS.

If SV does not increase sufficiently with dobutamine loading, the systolic flow reserve is considered to be impaired and the disease severity may be difficult to determine. In such cases, the AS severity should be assessed using findings from other modalities, such as calcification score assessment by computed tomography (CT). It has also been reported that patients with poor systolic flow reserve (SV increase <20%) have a higher risk of postoperative mortality compared to those with preserved systolic flow reserve (17). However, surgical or transcatheter AVR should be considered as it improves prognosis compared to conservative medical treatment alone regardless of the presence of flow reserve (18).

### 3.2. Exercise stress echocardiography in asymptomatic AS

Although the current ESC/EACTS guideline for the management of VHD mentions the incremental values of ESE, the ACC/AHA guideline does not specifically recommend ESE in patients with AS and its usefulness remains controversial. Of note, it is contraindicated in patients with symptoms definitively caused by AS due to the risk of major complications. Nonetheless, several reports demonstrated incremental values of ESE for asymptomatic AS patients. The main purposes of ESE for asymptomatic AS patients are to evaluate the essential risk of cardiovascular events and optimal timing for intervention. Elevated mean transvaluvlar PG (≥Δ18–20 mmHg) (19, 20), exercise induced pulmonary hypertension (Ex-PH) (≥60 mmHg on exertion) (21), and insufficient transvalvular flow reserve (22) have been indicated as markers of cardiovascular outcomes. Besides, patients' responses to exercise should be carefully monitored during ESE since establishing symptomatic status of AS patients can be challenging, particularly in elderly patients who may limit their own physical activity and their symptoms can be masked before exercise testing.

## 4. Mitral regurgitation

### 4.1. Primary mitral regurgitation

**Figure 5:**
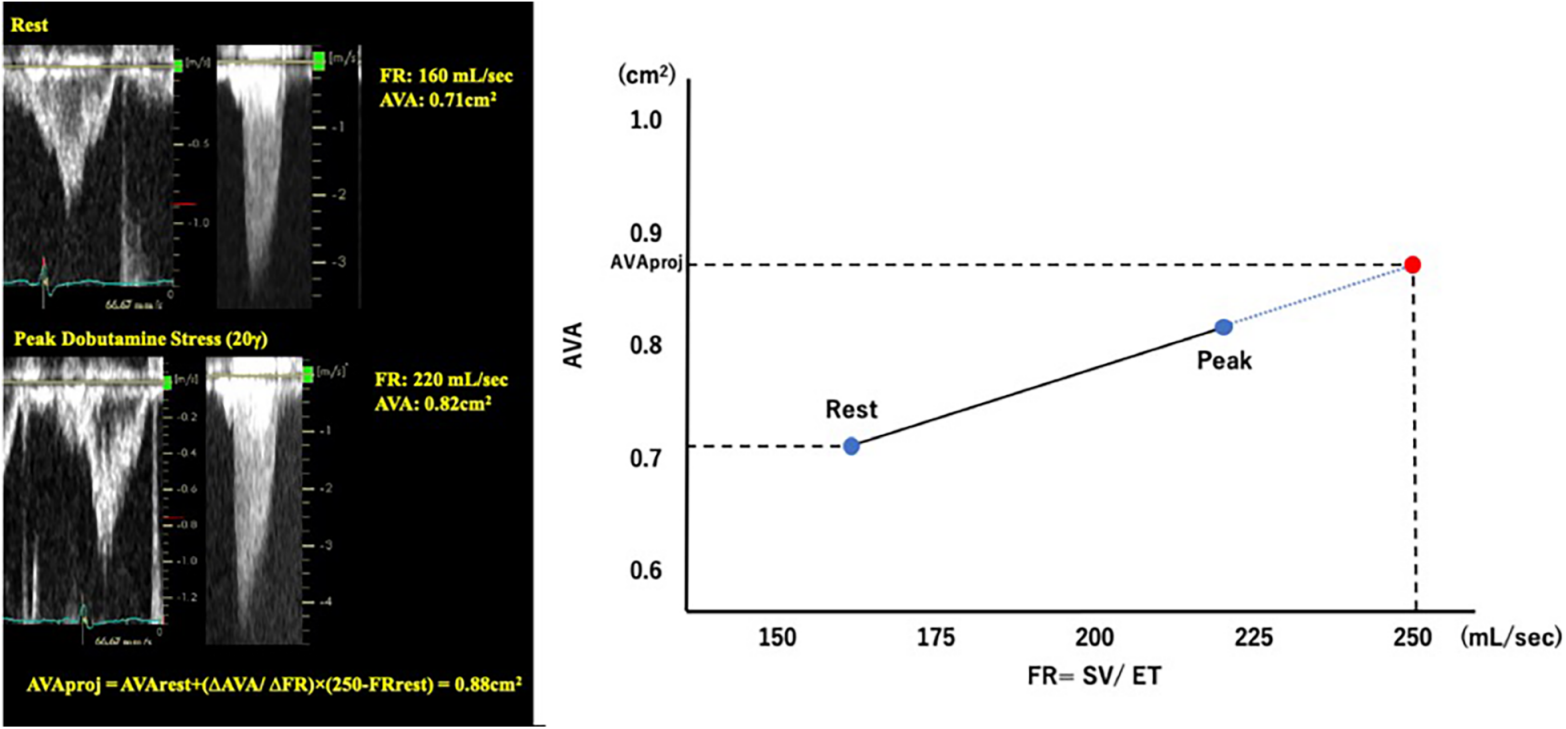
Exercise stress echocardiography for primary mitral regurgitation. Seventy-two-year-old woman, a case of primary MR due to anterior mitral leaflet prolapse. Dyspnea induced by 20 W exercise on a bicycle ergometer. ESE showed worsening of MR and pulmonary hypertension. EROA, effective regurgitation orifice area; ESE, exercise stress echocardiography; TRPG, tricuspid regurgitation pressure gradient.

Primary MR (PMR) refers to a systolic regurgitation from LV to left atrium (LA) due to organic abnormalities in mitral valve (MV) apparatus including leaflets, chordae tendineae and papillary muscles. Exercise testing incorporating hemodynamic assessment provides valuable clinical information, particularly for patients with asymptomatic chronic PMR or discordance between symptoms and regurgitation grade at rest. ESE, the most frequently used imaging modality, may help in comprehending the underlying pathophysiology of the condition. The prevalence of an increase in PMR (effective regurgitant orifice [ERO] ≥ Δ10 mm^2^ and regurgitant volume [RV] ≥ Δ10 ml) during exertion has been reported to occur in one-third of patients with PMR (23). Ex-PH, as indicated by estimated systolic pulmonary arterial pressure (SPAP) ≥ 60 mmHg, has also been regarded as an important finding (24) (Figure 5). These are regarded as reliable prognostic factors of cardiovascular risks. In addition, several new parameters such as absence of LV contractile reserve [ΔLVEF ≤ 5% and/or Δglobal longitudinal strain (GLS) ≤ 2%] (25, 26) and right ventricular systolic dysfunction [tricuspid annular plane systolic excursion (TAPSE) ≤ 18 mm] (27) during exertion are demonstrated their utilities in more recent studies. The accumulation of evidence may help to determine the optimal diagnosis, risk stratification, and timing of MV interventions in patients with PMR. Particularly in patients with these abnormal findings, early MV intervention may improve their prognosis. Prospective trials with large cohorts are required to establish the utility of SE and effectiveness of early intervention for PMR.

### 4.2. Secondary mitral regurgitation

Secondary MR (SMR) is a pathology that occurs with structurally normal MV apparatus and is caused by underlying dysfunction or abnormality in other multifactorial components of the heart. The severity of SMR is greatly affected by the hemodynamic condition (pre- and/or afterload) and therefore, the changes under stress condition are as dynamic in SMR as, or more so, than primary MR (28–31).

**Figure 6:**
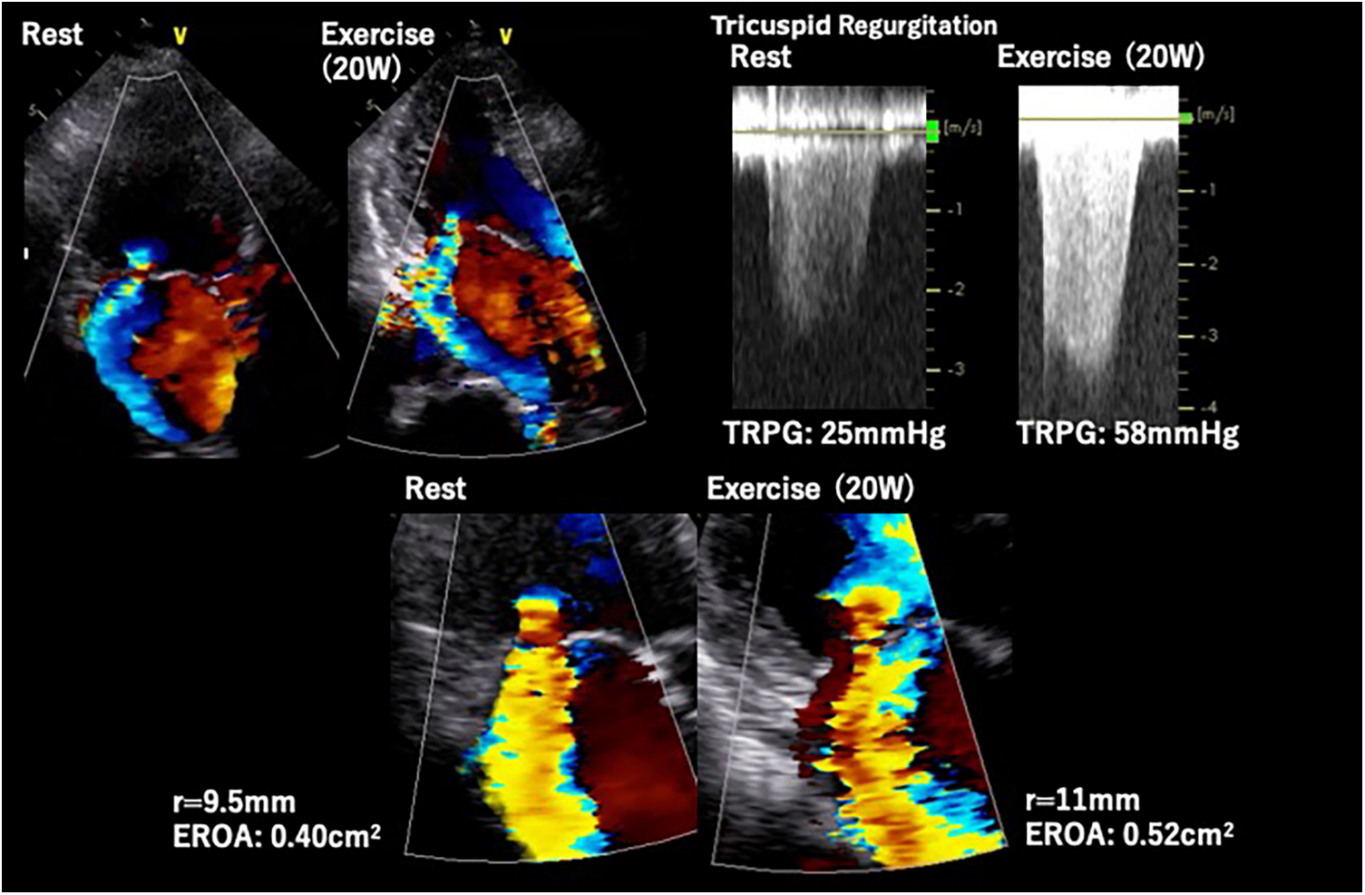
Exercise stress echocardiography for secondary mitral regurgitation. Seventy-eight-year-old man, a case of symptomatic secondary MR induced by non-ischemic cardiomyopathy. The MR severity changed from moderate to severe and quantitative parameters (EROA, RV, and SPAP) worsened during exercise. EROA, effective regurgitation orifice area; MR, mitral regurgitation; RV, regurgitation volume; SPAP, systolic pulmonary arterial pressure.

As shown in previous observational studies, a dynamic increase of SMR is recognized as a strong indicator of exercise capacity (30) and risk of cardiovascular events (28, 29). Moreover, Ex-PH (SPAP ≥ 60 mmHg during exercise) is associated with poor cardiovascular outcomes in patients with SMR as well as those with primary MR (32) (Figure 6).

Despite the development of diagnostic and assessment techniques, the treatment decision for SMR induced by stress remains controversial. For persistent severe SMR with heart failure with reduced ejection fraction (HFrEF), guideline-directed medical treatment (GDMT) such as beta-blockers, angiotensin-converting enzyme inhibitors (ACEi)/angiotensin II receptor blockers/angiotensin receptor-neprilysin inhibitors, mineralocorticoid receptor agonists, and sodium-glucose cotransporter 2 inhibitors should be administrated at their maximal dose (4, 5). LV reverse remodeling associated with these pharmacological treatments (33–36) improves persistent secondary MR with HFrEF and may also have a beneficial influence on exercise-induced SMR according to its mechanism (37).

Non-pharmacological treatments including cardiac resynchronization therapy (CRT) and transcatheter edge-to-edge MV repair (TEER) are also recommended for patients with persistent symptoms after appropriate coronary revascularization if applicable. CRT has been shown to improve LV function by suppressing LV dyssynchrony and subsequently reducing SMR not only at rest but also during exertion (38). Whereas, the prognostic significance of CRT administration has not been fully elucidated. A recent observational study suggests that TEER may be a safe and effective option for the treatment of exercise-induced SMR, albeit with a small patient sample (39).

## 5. Mitral stenosis

The hemodynamics of rheumatic mitral stenosis (MS) under stress condition have been extensively investigated over the past two decades (40). In some case with MS, exercise or pharmacological stress worsen the patients' symptoms due to a drastic increase in the LA pressure and SPAP, despite the relatively low PG at rest. Therefore, the usefulness of SE has been recognized for patients who have a discrepancy between the symptoms and the severity of MS at rest. In addition to unmask the symptoms under stress condition, SE provides hemodynamic information which can help determine intrinsic MS severity and the prognosis. The presence of a significant increase in mean transmitral PG (≥15 mmHg during exercise or ≥18 mmHg with dobutamine infusion) and/or Ex-PH (SPAP ≥ 60 mmHg during exercise) are considered as significant findings indicating poor prognosis in patients with rheumatic MS (41).

Moreover, an early increase in SPAP at low-level exercise regarded as a useful marker of symptoms induced by exercise in “asymptomatic” patients with rheumatic MS (42).

**Figure 7:**
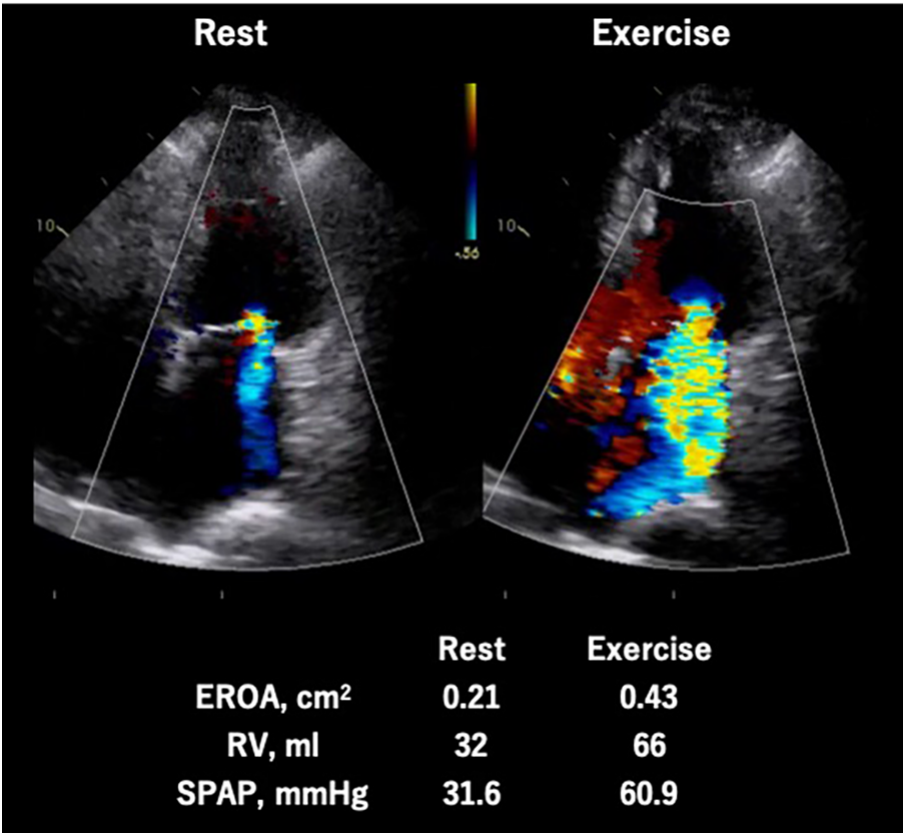
Exercise stress echocardiography for mitral stenosis. Eighty-four-year-old woman, a case of calcified severe MS confirmed by ESE. During exercise, MMPG increased from 5.7 mmHg to 24.9 mmHg and significant Ex-PH was observed. ESE, exercise stress echocardiography; MMPG, mean mitral pressure gradient; MS. mitral; stenosis; SPAP, systolic pulmonary arterial pressure.

Whereas, the utility of SE has not been fully elucidated for patients with degenerative MS, which is characterized by narrowing of mitral orifice due to the annular calcification (43). An observational study showed an increase in mean mitral PG and SPAP on exertion in degenerative MS patients with severe MAC similar to rheumatic MS (44) (Figure 7). However, degenerative MS may have a different hemodynamic response due to the presence of LV diastolic dysfunction and reduced LA compliance in relation to the typical elderly characteristic of patients with degenerative MS (43). Future studies are needed in this area to investigate the incremental benefits of SE in these patients and to help determine who will truly benefit from valvular interventions.

## 6. Post-operative assessment

Valvular replacement using prosthetic valve induces some degree of transvalvular PG at rest even in cases of normal valvular function. Therefore, SE may be useful in assessing not only native valvular disease but also prosthetic valve dysfunction, which is caused by degenerative stenosis and/or patients prosthesis mismatch (PPM). The evaluation on exertion or using dobutamine is recommended in patients with mild to moderately elevated transvalvular PG at rest, which referred as 20–40 mmHg in the aortic position and 5–10 mmHg in mitral position (41).

**Figure 8:**
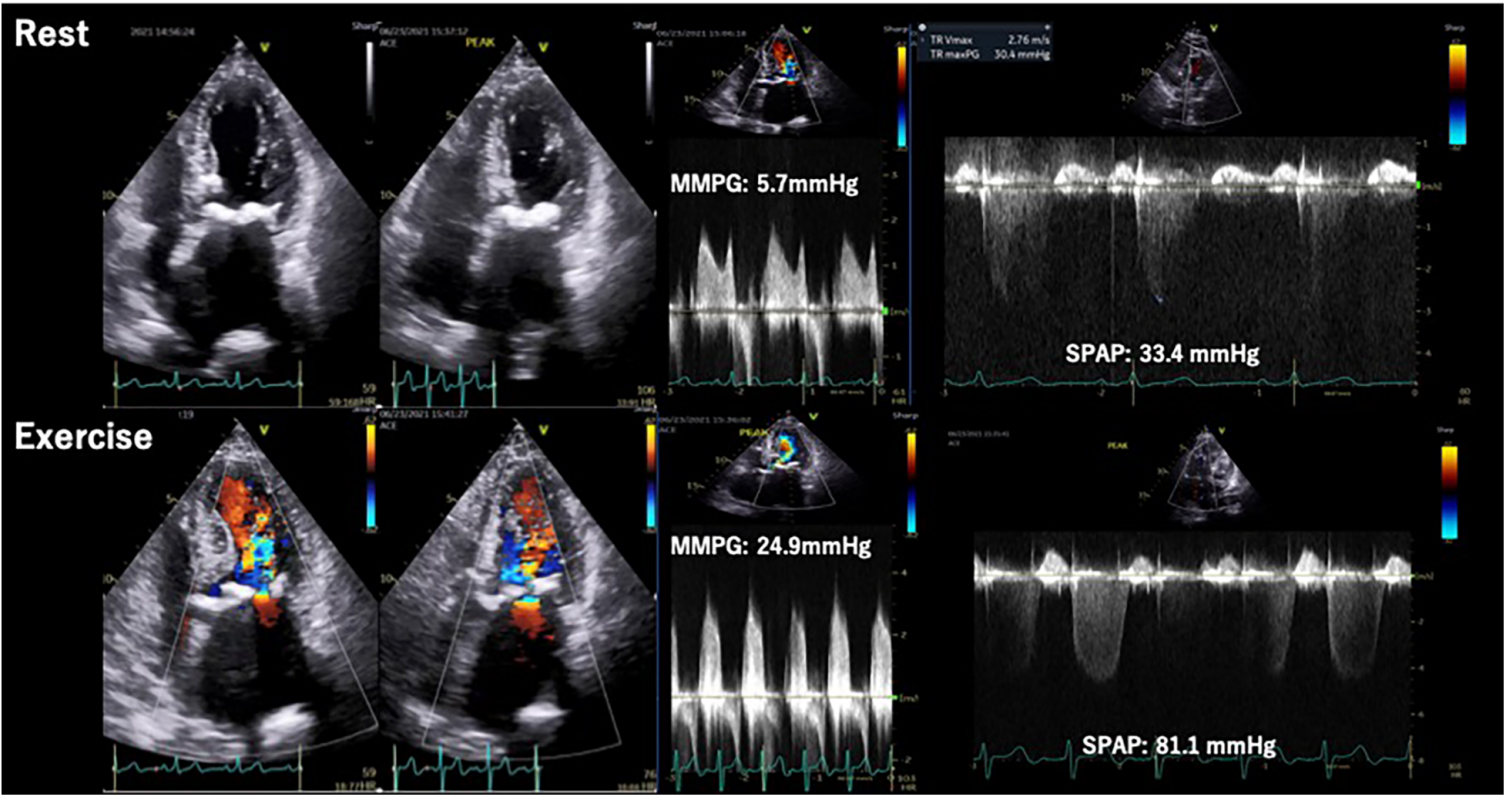
Stress echocardiography for prosthetic valve. Eighty-five-year-old man, a case of PPM after TAVI. Exercise stress induced increase of transprosthetic MPG, SPAP and worsening of diastolic dysfunction. MPG, mean pressure gradient; PPM, patient prosthesis mismatch; SPAP, systolic pulmonary arterial pressure; TAVI, transcatheter aortic valve implantation.

ESE is applied to clarify the association between exertional symptoms and hemodynamic prosthetic function. In addition to symptoms and Ex-PH, an increase in transprosthetic PG (>Δ20 mmHg for aortic position and >Δ10–12 mmHg for mitral position) is considered as significant finding indicating valve stenosis and/or PPM (45–47). In addition, a recent study reported PPM after TAVI is associated with disproportionate increase of transprosthetic PG on exertion and Ex-PH as shown in Figure 8 (48).

Low-dose DSE is also useful in assessing PPM and prosthetic valve dysfunction in low-flow conditions, especially when the discordance between the patients' symptomatic status and the resting hemodynamics are observed. Patients with low effective orifice area (EOA < 1.0 cm^2^) or abnormal Doppler velocity index (<0.35 for aortic position and >2.2 for mitral position) accompanied with low SV index (<35 ml/m^2^) should be evaluated with a low-dose DSE similar to LFLG-AS (41, 49).

In patients with normal flow reserve on exertion, a disproportionate increase in mean transvalvular PG (>Δ20 mmHg for aortic position and >Δ10 mmHg for mitral position) indicates the presence of true significant valve dysfunction or PPM. PPM is more frequent in patients with small prosthetic valves, referred to as <21 mm for the aortic position and <25 mm for the mitral position (13). Furthermore, degenerative prosthetic valve stenosis is observed in some cases. Whereas isolated PPM generally do not have EOA below the reference value for the implanted prosthetic valve, substantially smaller EOA than the reference value can be observed in patients with degenerative stenosis (41).

Functional mitral stenosis (MS) after mitral annuloplasty is reported to occur in up to 50% of post-operative patients and may induce several clinical problems including low exercise capacity and patients' quality of life (50). The transmitral flow rate frequently reduced in patients who underwent mitral annuloplasty as the significant proportion of patients has low cardiac function. Therefore, the severity of functional MS after mitral annloplasty may be underestimated at rest. ESE or DSE is currently recommended in symptomatic patients with mildly elevated transmitral gradient at rest (>3 mmHg). Absolute elevation of transmitral gradient ≥Δ7 mmHg with increasing SPAP (≥50 mmHg) are considered as abnormal findings for functional MS (41).

## 7. Others

The significance of SE in the other VHDs such as aortic regurgitation (AR) or tricuspid regurgitation (TR) are unclear compared to AS or MR.

Regarding AR, exercise testing may be useful for confirming diagnosis in severe AR patients with equivocal symptoms. Some echocardiographic parameters including LV contractile reserve (51), TAPSE (52), LV end-systolic volume index (53), and LV GLS (54) on exertion have been reported as prognostic parameters of AR. However, these findings were based on single-center observational studies and therefore, none of these parameters were included in current guidelines.

Evaluation of isolated TR using SE is also challenging since the development of TR is strongly affected by several confounders including preload, pulmonary arterial resistance, and right ventricular function. Moreover, the evidence of SE for primary TR is also missing.

LV diastolic function is also a factor that influence the result of SE for VHD. Especially in AS, the continuous increase of afterload due to stenotic aortic valve induces LV hypertrophy and may result to LV fibrosis. This is supported by a previous report showing that increasing LV filling pressure during exercise remains elevated in one third of patients after AV replacement (55). Furthermore, with regard to MR, left atrial dysfunction during exercise has also been reported (56, 57). Therefore, it is natural to consider that the LV diastolic dysfunction influences the symptoms through increased LV filling pressure in patients with significant VHD.

However, it would be difficult to distinguish clearly between the influence of VHD severity and LV diastolic dysfunction. Assessment of LV tissue using other imaging modality such as cardiac MRI may help to evaluate the extent of diastolic dysfunction by assessing LV fibrosis.

## 8. Conclusions

Given the development of therapeutic tools for VHD, accurate assessment of disease severity is becoming increasingly important in the management of patients with VHD. Echocardiographic assessment during exercise and pharmacological stress is safe and provides additive information that cannot be obtained at rest. In particular, cases with discrepancy between resting disease severity and symptoms should be assessed with SE in order to unmask the mechanism and hemodynamic characteristics. As shown in this review, different etiologies of VHD require different stress methods and different parameters to be measured on SE. SE should be appropriately indicated and understanding the hemodynamic influence under stress conditions is critical for accurate diagnosis and risk stratification of VHDs. Many studies have demonstrated the utility of SE in patients with various VHDs and therefore the application of SE in clinical practice may improve the management of VHD patients. However, most of the evidence is based on retrospective observational studies with small numbers of patients. Prospective studies with large numbers of patients and randomized trials are needed to confirm these findings regarding SE for VHDs.
